# The Adult Murine Intestine is Dependent on Constitutive Laminin-γ1 Synthesis

**DOI:** 10.1038/s41598-019-55844-x

**Published:** 2019-12-17

**Authors:** British Fields, Ann DeLaForest, Mark Zogg, Jennifer May, Catherine Hagen, Kristin Komnick, Jon Wieser, Alexander Lundberg, Hartmut Weiler, Michele A. Battle, Karen-Sue Carlson

**Affiliations:** 1 The Blood Research Institute of Wisconsin, part of Versiti, Milwaukee, Wisconsin USA; 2 The Medical College of Wisconsin, Department of Cell Biology, Neurobiology, and Anatomy, Milwaukee, Wisconsin, USA; 3 The Medical College of Wisconsin, Department of Pathology, Milwaukee, Wisconsin, USA; 4 The Medical College of Wisconsin, Department of Internal Medicine and Division of Hematology and Oncology, Milwaukee, Wisconsin USA

**Keywords:** Extracellular matrix, Stem-cell niche

## Abstract

Laminin-γ1 is required for early embryonic development; however, the need for laminin-γ1 synthesis in adulthood is unknown. A global and inducible mouse model of laminin-γ1 deficiency was generated to address this question. Genetic ablation of the Lamc1 gene in adult mice was rapidly lethal. Despite global Lamc1 gene deletion in tamoxifen-induced mutant mice, there was minimal change in total cardiac, pulmonary, hepatic or renal laminin protein. In contrast, laminin-γ1 was significantly depleted in the small intestines, which showed crypt hyperplasia and dissociation of villous epithelium from adjacent mesenchyme. We conclude that the physiologic requirement for laminin-γ1 synthesis in adult mice is dependent on a tissue-specific basal rate of laminin-γ1 turnover that results in rapid depletion of laminin-γ1 in the intestine.

## Introduction

Basement membranes are extracellular matrix (ECM) structures consisting of well-organized glycoproteins and glycosaminoglycans. These structures provide cell-scaffolding, facilitate adjacent cell-cell interactions, and bind cell-surface receptors to transmit environmental signals^1^. The laminin glycoproteins are requisite components of all basement membranes. Laminins are secreted as heterotrimeric glycoproteins, comprised of an α, β, and γ subunit^2–4^. At the cell surface, these three subunits self-assemble into a heterotrimer, which then forms a network of “ternary nodes”^5–12^. This is the initiating step in basement membrane formation as well as a structural and biochemical link to cell surface receptors, which in turn transmit survival and proliferative cues to membrane-adjacent cells^12,13^.

Of the 60 possible laminin heterotrimers, only 16 have been isolated from biological organisms, a majority of which contain the γ1 subunit^5,14^. Mouse embryos that are globally deficient in laminin-γ1 do not develop past embryonic day 5.5 (E5.5) after fertilization, and there are no reported human cases of complete congenital laminin-γ1 deficiency^3,4,15^. Embryologic and early post-natal deletion of laminin-γ1 in cell-specific conditional knockout mice has provided information about its function in the developing central and peripheral nervous systems and vasculature^16–20^. However, SNPs in or adjacent to the human LAMC1 gene that codes for laminin-γ1 have been identified by multiple GWAS studies as genetic risk factors for developing colorectal cancer^21–24^. Antibodies that react with laminin-γ1 have been detected in subsets of patients with pemphigoid^15,25–27^ and graft-versus-host disease^28^, and subepithelial basement membrane laminin-γ1 or laminin-γ1 containing heterotrimers are modified in the colons of patients with ulcerative colitis and Crohn’s disease^25,29–34^. However, these observations do not provide a mechanistic link between laminin-γ1 and the relevant clinical disorders, leaving open the question of whether laminin-γ1 expression in adulthood is a regulator of adult physiology. We generated a mouse model for temporal ablation of laminin-γ1 production in order to answer this question.

## Results

### Temporally controlled deletion of the LAMC1 gene In adult mice reveals organ-specific laminin-γ1 protein turnover

**Figure 1:**
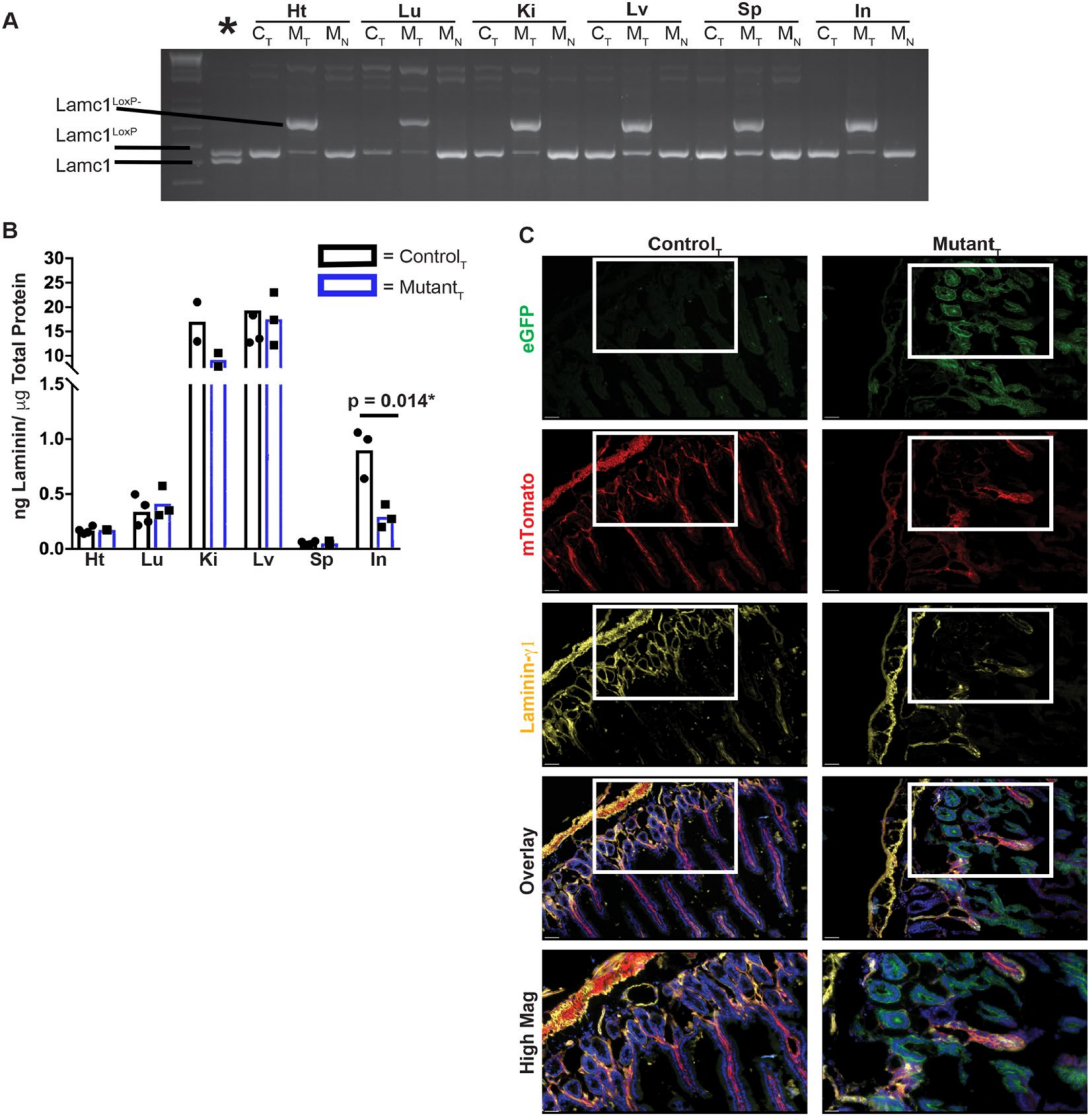
Conditional deletion of the Lamc1 gene in adult mice rapidly leads to laminin protein depletion in the small intestine. For (**A,B)**, tissues include heart (Ht), Lung (Lu), Kidney (Ki), Liver (Lv), Spleen (Sp) and Small Intestine (In). (**A**) Tissue from tamoxifen-treated control (C_T_), tamoxifen-treated mutant (M_T_), and untreated mutant (M_N_) were analyzed by PCR for the presence of wild type Lamc1, Lamc1^LoxP^, and recombined Lamc1^LoxP−^. The lane marked * contains tail DNA from a mouse with the genotype Lamc1^LoxP/+^. (**B**) Tissue lysates were analyzed by ELISA for total laminin protein concentration. Each data point is the average of two technical replicates from a single animal. (**C**) Immunofluorescent images of control and mutant small intestine that were probed with antibody against laminin-γ1 (yellow). Endogenous mTomato and eGFP are also shown. DAPI was used to label nuclei (blue). Magnification bars indicate 50 microns (lower magnification) and 10 microns (higher magnification). 10x and 40x with a 1.5 magnifier lenses were used to collect these images. Images are representative of the results obtained from immunofluorescent analysis of 3 tamoxifen-treated control and 4 tamoxifen-treated mutant mice.

Adult control (*LAMC1*^loxP/loxP^) and mutant (UBC-Cre-ERT2; *LAMC1*^loxP/loxP^) mice were treated with tamoxifen as described in *Methods*. Three weeks after the final tamoxifen injection, mice were euthanized. PCR was used to determine the recombination status of the *LAMC1*^loxP^ gene in heart, lung, liver, kidney, spleen and small intestine samples. The recombined *LAMC1*^loxP^ PCR-product (*LAMC1*^r^) was detected in all tamoxifen-treated mutant samples (M_T_) and in none of the tamoxifen-treated control samples (C_T_) (Fig. 1A). PCR of untreated mutant mice (M_N_) are also included and show no evidence of Lamc1^loxP^ gene recombination.

A sandwich ELISA was used to quantify the abundance of laminin in equivalent amounts of total protein from heart, lung, kidney, liver, spleen and intestine homogenates (Fig. 1B). In a majority of the organs sampled, the quantity of laminin protein did not significantly differ between tamoxifen-treated control and mutant mice. However, there was a significant reduction in total-laminin protein in the tamoxifen-treated mutant small intestine as compared with control.

The subepithelial laminin in adult intestinal crypts is laminin-211 and in the villi are laminin-332 and laminin-511^35,36^. Thus, laminin-ɣ1 is the exclusive sub-epithelial gamma-chain in intestine crypts and is a significant contributor to villous laminin. We used frozen tissue sections from control and mutant mice that also expressed the mTomato/eGFP reporter transgene^37^ to correlate areas of Cre-responsive gene recombination to laminin expression (Fig. 1C). In the absence of Cre recombinase activity, tissues express mTomato, which is lost with recombinase activity and replaced by eGFP expression. Tamoxifen-treated control mice expressed mTomato throughout the muscularis, crypts and villi, and minimal to no eGFP expression. Laminin-γ1 was detected in thin basement membranes surrounding crypts and extending through the duodenal villi. In the tamoxifen-treated mutants, mTomato fluorescence was visible in few villi and portions of the muscularis, while eGFP expression was robust in duodenal crypts villi. Laminin-γ1 immunoreactivity was restricted to mTomato^+^ villi and mTomato^+^ muscularis. The basement membranes adjacent to eGFP^+^ crypts and eGFP^+^ villi did not retain laminin-γ1 immunoreactivity. This indicates that Lamc1 gene recombination, and thus transition from mTomato to eGFP expression, corresponds with reduced laminin-γ1 protein in the adjacent basement membrane.

To compare *Lamc1* transcript abundance in both the epithelial and mesenchymal layers from control and mutant mice, duodenal epithelium was separated from mesenchyme, and RNA from each of the two fractions (epithelial and mesenchymal-enriched) were isolated and analyzed by qRT-PCR for *Lamc1* transcript (Table 1). The mesenchyme-enriched fractions from controls had higher *Lamc1* transcript levels than the epithelial fraction, and the more significant reduction of transcript was similarly in the mesenchyme of mutant mice. This finding is consistent with previously reported data^38^. Alternative laminin gamma subunit transcripts (i.e. -γ2 and γ3) were not upregulated in the mesenchyme of Lamc1 knockouts, although the laminin-γ2 transcript was upregulated in the epithelial fraction (Table 1, Fig. 1). Transcripts for α- and β laminin subunits were also compared (Table 1), as were laminin-α4 and laminin-α2 protein immunoreactivity (Supplemental Fig. 1).

**Table 1:** RT-PCR analysis of laminin subunit transcripts. * Indicates p < 0.05.

	Control + Tx (±SEM, n)	Mutant + Tx (±SEM, n)	p value
*Lamc1-Epi*	0.7239 ± 0.1507, n = 5	0.4477 ± 0.1047, n = 5	0.1708
*Lamc1-Mes*	13.12 ± 3.497, n = 9	2.984 ± 0.5256, n = 8	0.0165*
*Lamc2-Epi*	0.3355 ± 0.08577, n = 5	1.329 ± 0.4057, n = 6	0.0570*
*Lamc2-Mes*	2.237 ± 0.4147, n = 4	2.851 ± 1.042, n = 5	0.6348
*Lamc3-Epi*	0.002878 ± 0.001133, n = 2	0.003017 ± 0.001089, n = 2	ND
*Lamc3-Mes*	4.646 ± 2.199, n = 5	1.664 ± 1.528, n = 3	ND
*Lama1-Epi*	ND	ND	ND
*Lama1-Mes*	1.11 ± 0.3783, n = 4	1.364 ± 0.6026, n = 5	0.7479
*Lama2-Epi*	0.02868 ± 0.02555, n = 3	0.003025 ± 0.001197, n = 4	ND
*Lama2-Mes*	9.874 ± 3.751, n = 6	3.778 ± 1.679, n = 5	0.2007
*Lama3-Epi*	0.2167 ± 0.1197, n = 5	0.02989 ± 0.005951, n = 3	ND
*Lama3-Mes*	3.022 ± 0.667, n = 5	1.71 ± 0.6523, n = 5	0.1973
*Lama4-Epi*	0.05141 ± 0.01605, n = 4	0.4325 ± 0.4176, n = 5	0.4476
*Lama4-Mes*	25.53 ± 7.401, n = 6	5.337 ± 2.731, n = 5	0.0424*
*Lama5-Epi*	0.6793 ± 0.1252, n = 4	3.544 ± 2.921, n = 5	0.4160
*Lama5-Mes*	25.81 ± 8.991, n = 6	20.08 ± 12.49, n = 5	0.7121
*Lamb1-Epi*	0.2012 ± 0.08824, n = 5	8.044 ± 7.901, n = 5	0.3500
*Lamb1-Mes*	43.77 ± 12.29, n = 6	28.59 ± 13.01, n = 6	0.4160
*Lamb2-Epi*	0.1696 ± 0.04572, n = 4	0.02179 ± 0.006419, n = 4	0.0186
*Lamb2-Mes*	21.72 ± 17.39, n = 6	3.335 ± 1.125, n = 5	0.3644
*Lamb3-Epi*	1.006 ± 0.1636, n = 5	1.952 ± 0.5264, n = 6	0.1494
*Lamb3-Mes*	1.805 ± 0.4069, n = 5	1.845 ± 0.233, n = 5	0.9332

### Lamc1 gene deletion in adult mice is lethal

**Figure 2:**
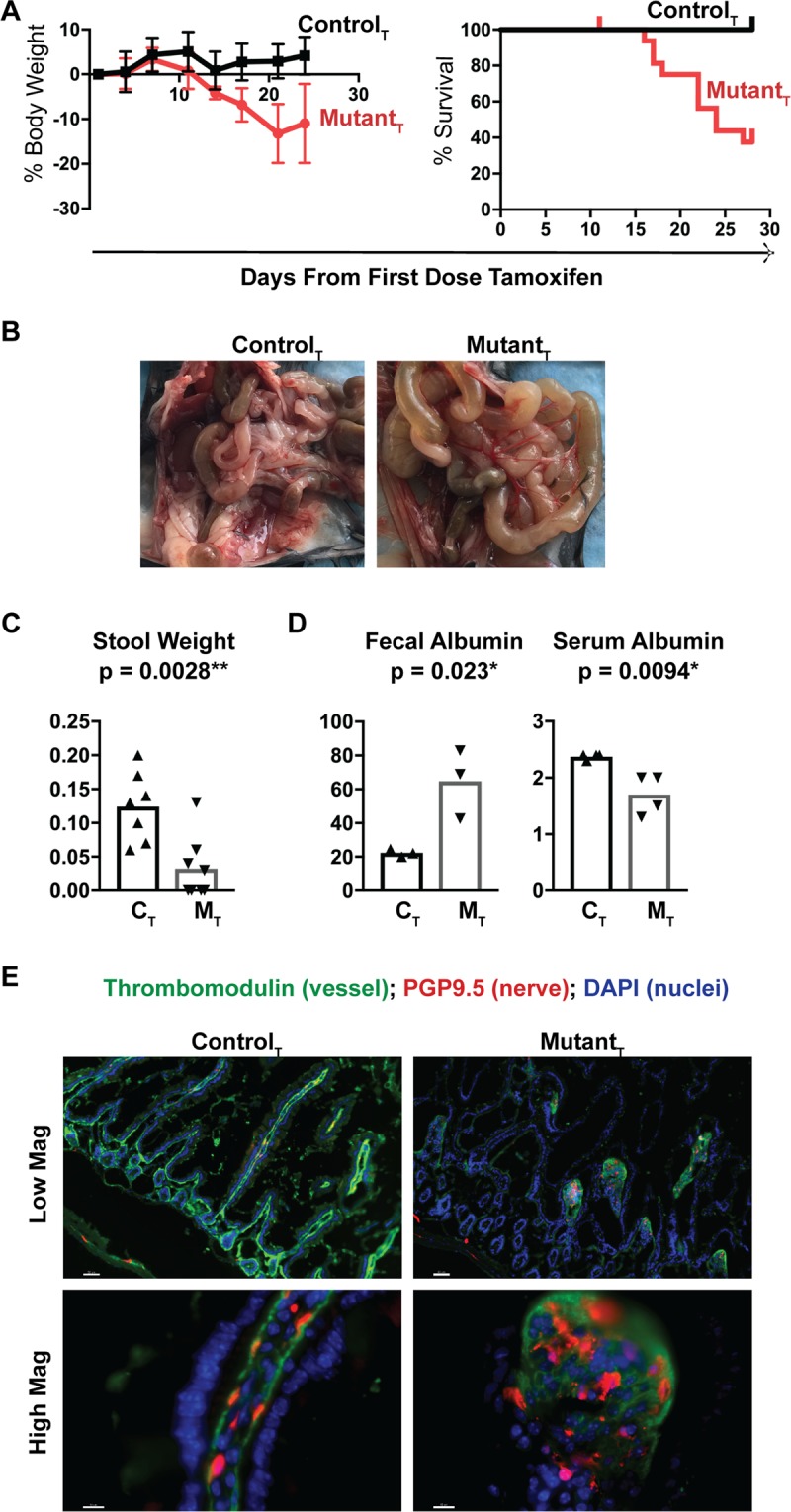
Conditional deletion of the Lamc1 gene in adult mice rapidly induces weight loss and morbidity/mortality that is due to gastrointestinal dysfunction. (**A**) Tamoxifen-treated control (black line) and tamoxifen-treated mutant (red line) mice were weighted the day of first tamoxifen injection, and throughout a three week period. Change in weight and survival are shown. Starting n = 10 for each cohort in weight graph, and mutant n = 17 and control = 10 for survival estimate. (**B**) Representative pictures of the intestines from tamoxifen treated control (n = 4) and mutant (n = 8) mice just after euthanasia. For (**C,D)**, tamoxifen-treated (C_T_), tamoxifen-treated mutant (M_T_). (**C**) Weight of stool collected over a 10-minute observation period and compared between genotypes. Each data point represents the results from a single mouse. (**D**) Fecal and serum albumin concentrations compared between genotypes. For panels C&D, each point represents the data from an individual animal. **(E)** Small intestine sections from C_T_ and M_T_ mice were immunostained with antibodies that recognize thrombomodulin (green) and PGP9.5 (red). Nuclei are stained blue with DAPI. Magnification bars are 50 microns in low magnification images, and 10 microns in high magnification images. 20x and 40x lenses with 1.5x magnifier were used to collect these images. Images are representative of the results obtained from immunofluorescent analysis of 3 tamoxifen-treated control and 4 tamoxifen-treated mutant mice.

Following tamoxifen administration, mutant and control mice were monitored with serial weight measurements and clinical observation. The control group maintained their body weight, while mutant animals progressively lost weight (Fig. 2A). Tamoxifen-treated mutant mice developed signs of distress, including ruffled fur appearance, hunched posture, lack of grooming behavior, and stunted growth. There was no evidence of overt hindlimb paresis (Supplemental Videos 1 Control & 2 Mutant), such as has been previously described in neuronal and Schwann cell-specific conditional Lamc1 knockout mice^16,17^. Clinical deterioration and weight loss was quantified using an IACUC-approved health metric scale, and mice falling below institutional acceptable scores were euthanized. This metric of morbidity was used to estimate survival from the first dose of tamoxifen administration (median survival of tamoxifen-treated control mice was unreached, and for tamoxifen-treated mutants was 24 days, p = 0.003; Fig. 2A). Once the time frame for 50 percent lethality was established, remaining control and mutant mice were euthanized. The tamoxifen-treated mice that survived to this end point had lost minimal weight, and by PCR evaluation of Lamc1^loxP^ recombination, had minimal gene recombination. There was no evidence of increased mortality in untreated mutant mice (*data not shown*). Based on this data, we concluded that the primary phenotype of adult laminin-ɣ1 deficient mice is rapid weight loss followed by mortality. Food and water intake were monitored in smaller cohorts of individually housed tamoxifen-treated control and mutant mice prior to and during a 21 day period inclusive of tamoxifen administration (starting n = 5 for each cohort; Supplemental Fig. 2**)**. No difference in food or water consumption was observed between cohorts.

### Lamc1 gene deletion in adult mice impairs gastrointestinal function and induces loss of serum proteins through the gut-vascular-barrier

Gross examination of small intestines just after euthanasia differentiated between tamoxifen-treated control and mutant tissues, the latter were pale and fluid-distended in comparison with controls (Fig. 2B). To estimate gastrointestinal function, we measured the weight of fecal pellets produced by a single mouse over 10 minutes (Fig. 2C). Laminin-deficient mice had less stool output by mass than did controls (control: 0.16 ± 0.016 grams, n = 4; mutant: 0.033 ± 0.036 grams, n = 4, p = 0.013). Stool pellets from both control and mutant mice were well-formed. No evidence of diarrhea or watery stool was noted.

Gut-vascular-barrier permeability defects cause serum proteins such as albumin to leak into the intestinal tract resulting in elevated fecal albumin and decreased serum albumin. To evaluate barrier function in the laminin-ɣ1-deficient mice, we measured stool and serum albumin content from both cohorts (Fig. 2D). The stool from laminin-deficient mice had a three-fold increase in fecal albumin and a small but statistically significant decrease in serum albumin in comparison with tamoxifen-treated control mice, consistent with a permeability defect.

We evaluated the lamina propria vasculature by immunofluorescent analysis of endothelium and adjacent nerve fibers. In healthy small intestine, the lamina propria microvasculature runs parallel to crypt and villous epithelium. Nerve fibers run adjacent to the microvessels through the center of the villi. We immunostained C_T_ and M_T_ small intestine for thrombomodulin (green) and PGP9.5 (red) to identify endothelial cells and nerve fibers, respectively (Fig. 2E). C_T_ microvasculature and nerve fibers followed the expected histologic organization. However, in M_T_ samples, thrombomodulin-positive endothelium and PGP9.5-positive nerve fibers were primarily detected in chaotic bundles at the interface between crypts and villi- without extension through the length of the villus lamina propria.

### Organ-system dysfunction in Lamc1 deleted adult mice is primarily restricted to the gastrointestinal track

Gross examination of other organs failed to identify other overt changes with the notable exceptions of reduced spleen and thymus size in the tamoxifen-treated mutant animals (*data not shown*). Serum electrolyte (Table 2) analysis was performed to screen for other organ-system specific patterns of illness in the laminin-depleted mice. Neither renal nor hepatic dysfunction were noted—specifically, renal indices of creatinine and blood urea nitrogen (BUN) were not elevated, and potassium, sodium, calcium, and phosphorous levels were unchanged between tamoxifen-treated control and mutant mice. Surrogates for hepatic function such as total bilirubin, ALT, AST and alkaline phosphatase also failed to differentiate between genotypes. Total serum protein and globulin differed between the two cohorts, with tamoxifen-treated mutant animals having lower serum concentrations of both. While other causes of mortality cannot be completely excluded, these findings identify a primary defect in gastrointestinal structure and function as the most likely cause of mutant mouse mortality and is consistent with the gastrointestinal tract as the primary site of rapid laminin-ɣ1 protein depletion.

**Table 2:** Serum chemistry analysis. * Indicates p < 0.05.

	Control + Tx (±SEM, n)	Mutant + Tx (±SEM, n)	p value
Alk Phos: U/L	89.25 ± 8.240, n = 4	91.00 ± 16.68, n = 4	0.9281
ALT (SGPT): U/L	23.75 ± 1.377, n = 4	61.75 ± 32.44, n = 4	0.2862
AST (SGOT): U/L	77.25 ± 20.56, n = 4	132.0 ± 66.99, n = 4	0.4643
Total Bilirubin: mg/dL	0.1250 ± 0.0250, n = 4	0.1000 ± 0.0, n = 4	0.3559
Total Protein: g/dL	4.400 ± 0.04082, n = 4	3.225 ± 0.3146, n = 4	0.0100*
Globulin: g/dL	2.025 ± 0.04787, n = 4	1.525 ± 0.1377, n = 4	0.0140*
BUN: mg/dL	29.25 ± 2.136, n = 4	30.50 ± 3.122, n = 4	0.7523
Creatinine: mg/dL	0.1750 ± 0.0250, n = 4	0.1000 ± 0.0, n = 4	0.0240*
Cholesterol: mg/dL	53.50 ± 4.592, n = 4	55.50 ± 4.113, n = 4	0.7566
Calcium: mg/dL	8.550 ± 0.08660, n = 4	8.333 ± 0.1202, n = 3	ND
Phosphorus: mg/dL	9.925 ± 0.3224, n = 4	8.925 ± 0.5154, n = 4	0.1511
TCO2: mmol/L	19.00 ± 3.028, n = 4	21.67 ± 3.844, n = 3	ND
Chloride: mmol/L	116.5 ± 0.9574, n = 4	120.3 ± 2.016, n = 4	0.1438
Potassium: mmol/L	4.200 ± 0.3416, n = 4	5.125 ± 0.4366, n = 4	0.1462
Sodium: mmol/L	149.5 ± 1.190, n = 4	153.0 ± 2.041, n = 4	0.1891

### Lamc1 deletion in adult mice induces duodenal crypt hyperplasia, villus elongation, and dissociation of epithelium from adjacent mesenchyme

**Figure 3:**
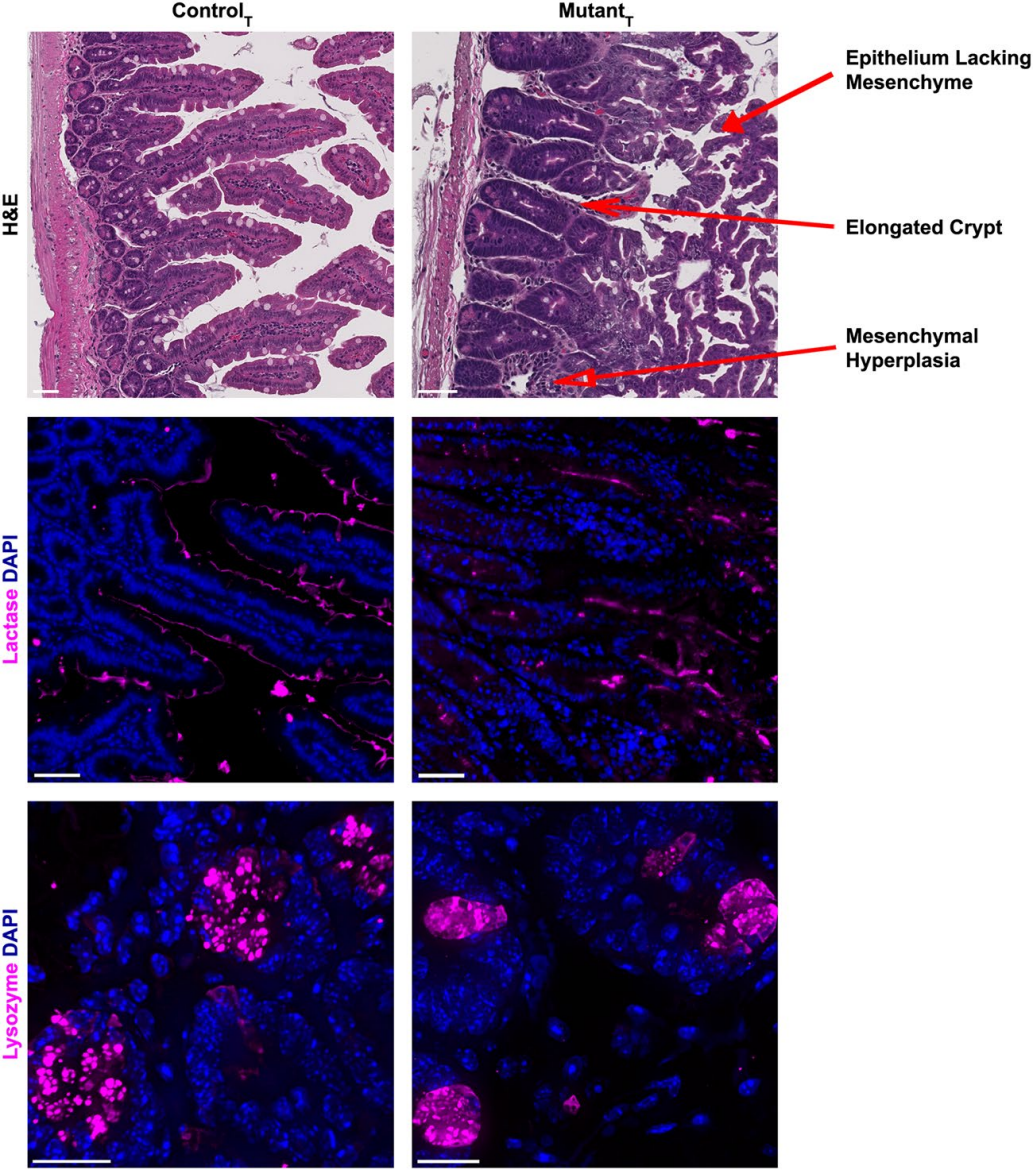
Laminin-ɣ1 deficiency causes intestinal epithelial hyperplasia. Hematoxylin and eosin stained paraffin-embedded tissue sections from tamoxifen-treated control and mutant duodenum. Magnification bars indicate 50 microns. Tamoxifen treated control and mutant paraffin-embedded duodenum sections were stained with antibodies against lactase or lysozyme (magenta). DAPI (blue) was used to label nuclei. Magnification bars indicate 50 microns for panels denoting lactase staining, and 20 microns for those showing lysozyme immunoreactivity. Lysozyme images are a maximum intensity projection image derived from a z-stacked image obtained with a 100x oil lens. Images are representative of the results obtained from immunofluorescent analysis of 3 tamoxifen-treated control and 4 tamoxifen-treated mutant mice.

Histologic examination of duodenum from the laminin-deficient mice using hematoxylin and eosin stain revealed epithelial hyperplasia, with development of complex villous architecture and crypt hyperplasia (Fig. 3). In the laminin-deficient mice, the distal-most villi lacked associated mesenchyme, although mesenchyme remained and was hyperplastic adjacent to the point of transition between crypt and villous epithelium. To determine whether both absorptive and secretory epithelia were affected by laminin-ɣ1 deficiency, duodenum sections were stained with antibodies against lactase and lysozyme, and epithelial-specific RNA transcript levels were compared. Mutant intestines had reduced lactase immunoreactivity, and abnormally diffuse lysozyme positivity rather than the concentrated granular staining noted in control samples (Fig. 3). Comparison of duodenal epithelial transcript levels between control and mutant mice did not show a consistent pattern of dysregulation, although lactase transcript was reduced in the mutants, consistent with the observed reduction in immunoreactive lactase protein (Table 3).

**Table 3:** RT-PCR Analysis of Epithelial and Mesenchymal Transcripts.

	Control + Tx (±SEM, n)	Mutant + Tx (±SEM, n)	p value
*Lgr5-Epi*	0.2864 ± 0.07627, n = 9	0.0508 ± 0.01402, n = 10	0.0053**
*Olfm4-Epi*	15.15 ± 4.903, n = 9	3.761 ± 1.336, n = 10	0.0311*
*Tert-Epi*	0.2001 ± 0.03286, n = 8	0.07712 ± 0.01662, n = 7	0.0071**
*HopX-Epi*	11.66 ± 2.426, n = 5	9.494 ± 2.167, n = 5	0.5245
*Lactase-Epi*	4.366 ± 1.627, n = 5	0.1924 ± 0.1606, n = 4	0.0587
*Muc2-Epi*	14.78 ± 4.279, n = 6	28.44 ± 18.41, n = 6	0.4866
*Lysozyme-Epi*	94.24 ± 46.41, n = 6	67.62 ± 39.26, n = 6	0.6709
*CgA-Epi*	2.54 ± 0.768, n = 9	0.6836 ± 0.0898, n = 10	0.0214*
*NGN3-Epi*	1.852 ± 0.8388, n = 6	0.9663 ± 0.7061, n = 5	0.4514
*Gata4-Epi*	149.5 ± 1.190, n = 4	153.0 ± 2.041, n = 4	0.1891
*Gata6-Epi*	8.550 ± 0.08660, n = 4	8.333 ± 0.1202, n = 3	ND
*Ihh-Epi*	0.2412 ± 0.05371, n = 7	0.08471 ± 0.01031, n = 10	0.0039**
*Patched1-Mes*	5.71 ± 1.578, n = 9	1.863 ± 1.002, n = 7	0.0755
*Abcg5-Epi*	4.400 ± 0.04082, n = 4	3.225 ± 0.3146, n = 4	0.0100*
*Abcg8-Epi*	29.25 ± 2.136, n = 4	30.50 ± 3.122, n = 4	0.7523
*Ada-Epi*	35.88 ± 10.76, n = 6	36.81 ± 15.29, n = 6	0.9614
*Akr1b8-Epi*	23.75 ± 1.377, n = 4	61.75 ± 32.44, n = 4	0.2862
*Apoa4-Epi*	4.200 ± 0.3416, n = 4	5.125 ± 0.4366, n = 4	0.1462
*ApoB-Epi*	89.25 ± 8.240, n = 4	91.00 ± 16.68, n = 4	0.9281
*ApoC2-Epi*	50.03 ± 17.52, n = 6	83.5 ± 27.38, n = 6	0.3275
*ApoC3-Epi*	21.29 ± 7.974, n = 6	19.8 ± 8.618, n = 5	0.9019
*Asah2-Epi*	3.839 ± 1.392, n = 5	1.091 ± 0.4592, n = 4	0.1355
*Cck-Epi*	0.1250 ± 0.0250, n = 4	0.1000 ± 0.0, n = 4	0.3559
*CD36-Epi*	0.3076 ± 0.1051, n = 4	0.1526 ± 0.04739, n = 4	0.2274
*Cftr-Epi*	10.03 ± 1.854, n = 4	4.231 ± 0.4631, n = 5	0.0116*
*Dnase1-Epi*	4.018 ± 1.422, n = 6	0.04963 ± 0.02351, n = 5	0.0325*
*Enpp7-Epi*	0.1001 ± 0.02252, n = 4	0.009414 ± 0.007271, n = 3	ND
*Fabp2-Epi*	116.5 ± 0.9574, n = 4	120.3 ± 2.016, n = 4	0.1438
*Hsd17b6-Epi*	7.889 ± 2.534, n = 5	3.785 ± 2.251, n = 4	0.2778
*Pdx1-Epi*	2.025 ± 0.04787, n = 4	1.525 ± 0.1377, n = 4	0.0140*
*Slc2a1-Epi*	2.236 ± 0.7246, n = 5	4.314 ± 0.5867, n = 5	0.0564
*Slc2a2-Epi*	53.50 ± 4.592, n = 4	55.50 ± 4.113, n = 4	0.7566
*Slc2a5-Epi*	9.925 ± 0.3224, n = 4	8.925 ± 0.5154, n = 4	0.1511
*Slc2a8-Epi*	0.3324 ± 0.08289, n = 4	0.5209 ± 0.3911, n = 5	0.6875
*Slc5a1-Epi*	15.69 ± 4.248, n = 5	6.339 ± 2.323, n = 4	0.1170
*Slc5a11-Epi*	1.191 ± 0.2438, n = 4	0.1546 ± 0.1244, n = 4	0.0091**
*Sst-Epi*	0.1750 ± 0.0250, n = 4	0.1000 ± 0.0, n = 4	0.0240*
*Ugt2a3-Epi*	6.886 ± 1.61, n = 5	3.701 ± 1.32, n = 5	0.1646

* Indicates p < 0.05 and ** Indicates p < 0.01.

### Intestinal epithelial cell proliferation is augmented in laminin-γ1 deficient intestines

**Figure 4:**
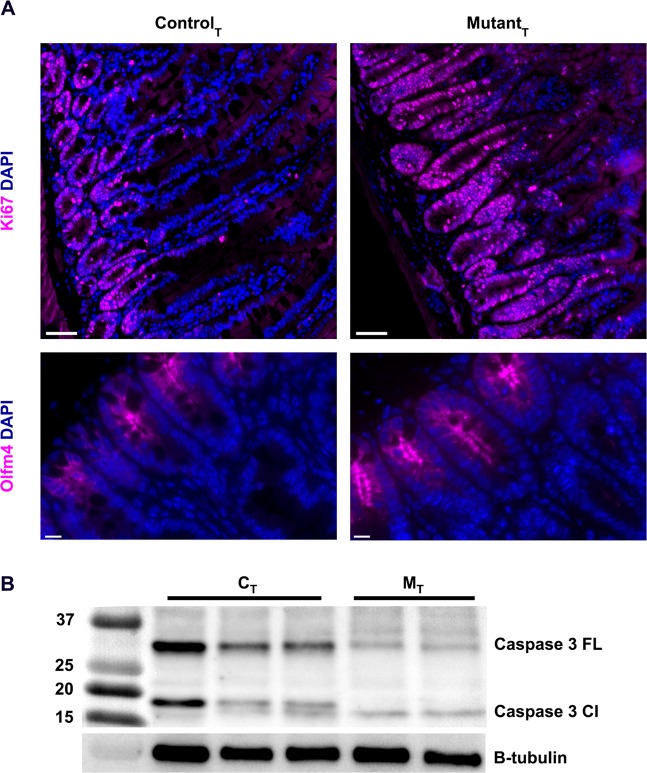
ISPC homeostasis is dysregulated in laminin-ɣ1 deficient intestines. (**A**) Tamoxifen treated control and mutant paraffin-embedded duodenum sections were stained with antibodies against ki67 or Olfm4 (magenta). DAPI (blue) was used to label nuclei. Magnification bars indicate 50 microns for Ki67 and 10 microns for Olfm4. Images are representative of the results obtained from immunofluorescent analysis of 3 tamoxifen-treated control and 4 tamoxifen-treated mutant mice. **(B)** Western blot of small intestine from tamoxifen-treated control and mutant mice for caspase 3 expression. Each lane derived from an individual animal. Full western blots for Caspase 3 and B-tubulin are shown in Supplemental Fig. 3.

Laminin-ɣ1-deficient intestines had elongated crypts and expanded tubular villi, suggestive of altered epithelial proliferation. To determine whether this was the case, we examined tissue sections using an antibody against nuclear Ki67, which identifies cells in active cell cycle (Fig. 4A). Control samples had a small number of Ki67^+^ cells, the localization of which was limited to the transit amplification zone and crypt base. However, in the mutant samples, the entire length of the elongated crypts was positive for nuclear Ki67, and ectopic foci of Ki67^+^ nuclei were also noted in the villi. We then asked whether apoptotic cell death was increased in the laminin-ɣ1 deficient intestines by immunoblot of epithelial cell lysates for caspase 3 (Fig. 4B, Supplemental Fig. 3). In the laminin-ɣ1 deficient samples, neither total nor cleaved caspase 3 were increased above control levels, and thus did not show evidence of increased apoptosis.

To determine whether the stem cell pool was impacted by expansion of the Ki67^+^ transit amplification zone, we compared stem cell transcript levels between control and mutant mice. In epithelium isolated from laminin-γ1 deficient mice, Lgr5^39–41^, Olfm4^42,43^ and Tert^44^ transcripts were significantly reduced, while HopX transcripts^45^ was relatively preserved (Table 3). Interestingly, Olfm4 protein expression did not differ between the two cohorts (Fig. 4A). These data are consistent with a significant reduction in the Crypt Base Columnar stem cell pool (Lgr5 and Olfm4). The +4 label retaining quiescent stem cell pool (Tert and HopX) is only somewhat reduced. Neither stem cell population is expanded, leading us to conclude that the Ki67+ cells observed in Fig. 4A are not intestinal stem cells.

### Epithelial Indian hedgehog transcript is reduced in laminin-ɣ1 deficient intestines

The hedgehog pathway is an important regulator of epithelial-mesenchymal homeostasis in the gut. Developmental epithelial Ihh deficiency (Villin-Cre; Ihh^loxP/loxP^) leads to early mortality, crypt hyperplasia, transit amplification zone elongation, and mesenchymal expansion at villus bases^46^. These morphologic changes are similar to what we observed in the laminin-ɣ1 deficient mutants. To further define the degree to which gut homeostasis is dysregulated in the laminin-γ1 deficient mice, we asked whether hedgehog pathway transcripts were altered in the laminin-γ1 depleted small intestines.

Mutant epithelium had a 2.8-fold reduction in *Ihh* transcript compared with controls (control = 0.2412 ± 0.05371, n = 7; mutant = 0.08471 ± 0.01031, n = 10; p = 0.0039**; Table 3). Ihh not only binds to its receptor Patched1 on mesenchymal cells, it regulates its transcription. Reduced *Ihh* transcript corresponded with a trend towards reduced *Patched1* transcript levels in mesenchyme from the same animals (control = 5.71 ± 1.578, n = 9; mutant = 1.863 ± 1.002, n = 7, p = 0.0755; Table 3).

## Discussion

The laminin-γ1 subunit is the most prevalent gamma subunit in laminin heterotrimers isolated from living tissue. Because of the early lethality of laminin-γ1 deficiency in embryologic development, its function in adult physiology is unknown. While laminin-ɣ1 is present in most adult tissues, protein turnover and thus need for active and constitutive synthesis was primarily noted in the gastrointestinal tract. In the small intestine, *Lamc1* gene recombination leads to reduced abundance of mesenchymal *Lamc1* gene transcript, and a significant reduction in laminin-ɣ1 protein expression. In heart, lung, kidney, liver and spleen, minimal protein reduction was evident three weeks post-induction, suggesting tissue-specific equilibrium of laminin-ɣ1 protein synthesis and degradation.

These results indicate that laminin-γ1 protein is actively turned over and replaced in the adult gastrointestinal tract. In the absence of nascent protein synthesis, the laminin-γ1 content of the small intestine is reduced within three weeks of gene recombination. This has a significant effect on intestinal histology and function. Although both epithelial and mesenchymal compartments are hyperplastic, it is neither coordinated nor functional. Mesenchymal structures, including disorganized neurovascular bundles expand but fail to extend past the villous bases, while numerous villous epithelium stream away from their mesenchymal support and blood supply. These structural changes underlie the gut-vascular barrier dysfunction and increased morbidity induced by *Lamc1* gene deletion in the adult mice. The primary source of laminin-γ1 appears to be the mesenchyme, with relatively minimal *Lamc1* transcript derived from the epithelium. This is consistent with Li *et al*.^38^ whose gene expression analysis comparing epithelial and mesenchymal expression patterns showed similar results.

Reduced epithelial transcription of *Ihh* is further supportive of our conclusion that epithelial and mesenchymal homeostasis is disrupted in the laminin-γ1 depleted intestines. It is tempting to speculate that this may be more than a marker of disequilibrium, and may in fact be a significant contributor to the mechanism by which laminin-γ1 alters epithelial proliferation. The laminin-ɣ1 deficient small intestines described here, and Ihh^46,47^ deficient small intestines have several morphologic and biochemical similarities. Epithelial Ihh deficiency (Villin-Cre; Ihh^Loxp/Loxp^) is lethal during early postnatal development because of gastrointestinal dysfunction and malnutrition. These mice also have crypt hyperplasia and reduced transcript levels for extracellular matrix proteins, including the *lamc1* transcript^46^.

Importantly, our results indicate that laminin-γ1 synthesis and degradation in the adult intestinal stem cell niche are actively regulated. Within the limited time frame dictated by the onset of gastrointestinal morbidity following tamoxifen induction of Lamc1 gene deletion in these mice, there were minimal changes in the laminin content or function of the other organ systems we examined. This leads us to conclude that the synthesis and turnover of laminin-γ1 is a context-dependent regulator of gastrointestinal homeostasis. While renal and hepatic function did not change with Lamc1 gene deletion in adult animals, it will be informative to determine whether other adult stem cell niches that actively and constitutively produce lineage-defined cells, such as the bone marrow, are similarly dependent on laminin homeostasis.

The correlation between laminin-γ1 homeostasis and human diseases of adulthood is also unknown. It is certainly noteworthy that LAMC1 SNPs and mutations have been identified as predisposing factors in the development of colorectal malignancy^21–24^. The rapid development of epithelial hyperplasia in our laminin-γ1-deficient mice provides some functional information relevant to these GWAS studies and leads us to ask whether perturbation of laminin-γ1 homeostasis is a specific contributor to the evolution of gastrointestinal malignancy.

## Materials and Methods

### Inducible and conditional mutant mouse generation

Mouse husbandry and research protocols were carried out in accordance with protocol AUA00003140, which was approved by The Medical College of Wisconsin’s Institutional Animal Care and Use Committee (IACAUC). C57BL/6 mice homozygous for the LoxP-flanked *Lamc1* gene (*Lamc1*^LoxP^) which encodes for laminin-γ1 protein^16–18,48^ (Strain: B6.129P2-*Lamc1tm1Strl*/J; Jackson Laboratories, Bar Harbor, ME) were bred with C57BL/6 mice that express the UBC-Cre-ERT2^49,50^ transgene (Strain: B6.Cg-Tg(UBC-cre-ERT2)1Ejb/J; Jackson Laboratories, Bar Harbor, ME). Genotypes were verified by isolation of genomic DNA and PCR for Cre and *Lamc1*^*LoxP*^ as previously described^16,17,37^. All founder animals had been backcrossed at least nine generations onto the C57Bl/6J background. Mice with the genotype UBC-Cre-ERT2^+/−^; *Lamc1*^*LoxP/LoxP*^ are hereafter referred to as *mutant*, and *Lamc1*^*LoxP/LoxP*^ are referred to as *control*. In specified experiments, *mutant* and *control* mice also expressed the reporter ACTB-tdTomato,-EGFP transgene^37^ (Strain: B6.129(Cg)-*Gt(ROSA)26Sor*^*tm4(ACTB-tdTomato,-EGFP)Luo*^/J; Jackson Laboratories, Bar Harbor).

### Tamoxifen-induction of Lamc1^fl^ recombination

Tamoxifen (Sigma-Aldrich, St Louis, MO) was solubilized in corn oil via overnight incubation at 37 °C with agitation. 1 mg was administered via intraperitoneal injection once-daily for four days (4 mg total per mouse). *Lamc1*^*LoxP*^ recombination was verified by PCR for the post-recombination gene product (*Lamc1*^r^) using the following primer sets (1: GCCTTCTATCGCCTTCTTGAC; 2: AAAGAAGCAGAGTGTGGGGG, and 3: TGGCCTTTTCAACCCTGGAA).

### Tissue lysate preparation for Laminin ELISA

Tissues were homogenized using a IKA ULTRA-TURRAX T8 homogenizer in an extraction buffer containing 100 mM Tris, pH 7.4, 150 mM NaCl, 1 mM EGTA, 1 mM EDTA, 0.5% Sodium deoxycholate, 1 mM Sodium Orthovanadate (Sigma, St Louis MO) or Halt Phosphatase inhibitor cocktail (Thermo Fisher, Walthm, MA), and protease inhibitors (EDTA-free protease inhibitor cocktail, Sigma, St Louis MO). Samples were agitated at 4 °C for 2 hours and then centrifuged for 20 minutes at 13,000 rpm. The total protein content of the resultant supernatant was quantified via BCA protein assay (#23227, Thermo Fisher Scientific, Waltham, MA).

### ELISA

The laminin concentration of tissue lysates was quantified relative to total protein using a total Laminin sandwich ELISA kit (#ab119572; Abcam, Cambridge, MA).

### Epithelium and mesenchyme tissue fractionation

Intestinal epithelium and mesenchyme were separated by incubation of tissue fragments in ice cold BSS buffer (1 mM KCl, 96 mM NaCl, 27 mM Sodium Citrate, 8 mM KH2PO4, 5.6 mM Na2HPO3, 15 mM EDTA) containing protease and phosphatase inhibitors at 4 °C with vigorous shaking for 30 minutes. Sheets of mesenchyme are then removed with forceps.

### Western blot

Equivalent amounts of protein from each sample type were heated for 10 minutes to 90 °C in reducing buffer and then analyzed by Tris-Glycine SDS-PAGE (BioRad; Hercules, CA) followed by semi-dry protein transfer onto PVDF membrane (BioRad; Hercules, CA). Blots were blocked with Tris Buffered Saline with 0.1% tween (TBST) and 5% non-fat dry milk overnight at 4 °C, followed by another overnight incubation at 4 °C with one of the following primary antibodies diluted into TBST: anti-Caspase-3 (ab13847, Abcam; Cambridge, MA), anti-β-Tubulin (#PA1-41331, Thermo Fisher Scientific, Waltham, MA; 1:1000). Secondary antibody was anti-rabbit IgG HRP (#sc-2357, Santa Cruz Biotechnology, Dallas, TX), and SuperSignal Western Blotting Substrates (Dura and Femto; Thermo Fisher Scientific; Waltham, MA) were used for protein detection. Chemiluminescence was detected using a GE ImageQuant LAS 4000, and ImageJ was used to quantify band intensity from the digital images. Full images of cropped western blots is included in Supplemental Fig. 3.

### Tissue preparation for histological characterization

Following euthanasia according to IACUC approved animal use protocol, tissues were either immediately flash-frozen in OCT (used for imaging laminin-γ1), or post-fixed in 10% formalin followed by paraffin embedding or sucrose cryoprotection and/or OCT embedding. Frozen sections were prepared using a Leica cryostat, equipped with a CryoJane tape mechanism. Hematoxylin and Eosin staining was performed by the Blood Research Institute histology core according to standard protocols. H&E stained duodenum sections were analyzed by a clinical gastrointestinal pathologist who was blinded to genotype at the time of slide analysis.

### Immunohistochemistry and immunofluorescence

Paraffin embedded sections were deparaffinized and rehydrated, followed by heat-mediated antigen retrieval in Epitope Unmasking Solution (BioWorld; Dublin, OH). Non-specific antibody binding was blocked using No Protein Block (VWR, Radnor, PA), FC Block (Innovex, Richmond, CA) and Horse Serum (Vector Labs, Burlingame, CA) in Tris-Buffered Saline. The same protocol was used for frozen sections beginning with the blocking step. Experiments using isotype-specific or species specific non-immune IgG in lieu of primary antibody were used as negative controls for all immunofluorescence experiments. Representative images are shown in each figure, with the number of biological replicates for each group detailed in the figure legends. *Primary antibodies:* Anti-laminin-γ1 (MAB1914B [A5 clone]; EMD Millipore, Burlignton, MA), Anti-laminin-α4 (AF3837; R&D systems, Minneapolis, MN); Anti-laminin-α2 (L0663 [4H8-2 clone]; Sigma, St Louis, MO), Anti-Olfm4 (NBP2-24535SS; Novus, Centennial, CO), Anit-Ki-67 (#ab15580; Abcam, Cambridge, MA), Anti-lysozyme (rabbit-derived, provided by Dr. Tomas Ganz, UCLA to MAB), thrombomodulin (AF3894; R&D systems, Minneapolis, MN), PGP9.5 (ab108986, Abcam, Cambridge, MA), and lactase (SC-240614, Santa Cruz Biotechnology, Dallas, TX). *Secondary antibodies:* Alexa-488 conjugated anti-Rabbit IgG, rat IgG and anti-Goat IgG, and Alexa-594 conjugated anti-Rabbit IgG, rat IgG and Goat IgG were from (Invitrogen/Life Technologies; Waltham, MA). Alexa -647 conjugated anti-Goat IgG, anti-Rabbit IgG, and anti-Rat IgG were from Jackson Immuno Research, West Grove, PA. Immunofluorescent staining was imaged using an inverted Nikon Ti-2 E high-speed motorized microscope equipped with the following objectives: CFI Plan APO Lambda 20× (MRD00205), CFI Plan APO Lambda 40× (MRD00405), and CFI Plan APO Lambda 100x oil (MRD01905) objectives. Post-acquisition image processing was performed with Nikon Elements for lysozyme images, Nikon Deconvolution packages. All images were formatted using Imaris software. Image processing parameters were applied equivalently to paired mutant and control images.

### Fecal albumin quantification

The stool albumin content was determined by solubilizing 20 mg of stool in 300 µl of cold dilution buffer (included in Mouse Albumin ELISA Kit, E99-134, Bethyl Laboratories, Montgomery, TX) for 15 minutes on ice, followed by vortex and centrifugation at 13,000 rpm for 5 minutes. Supernatant was used and analyzed by ELISA according to manufacturers protocol (Mouse Albumin ELISA Kit, E99-134, Bethyl Laboratories; Montgomery, TX).

### Plasma chemistry measurement

Peripheral blood was collected via inferior vena cava draw at the time of euthanasia. Plasma was prepared and then flash frozen. Analysis was performed by IDEXX (West Sacramento, CA).

### RT-PCR analysis

RNA was extracted via Trizol and Trizol LS reagents (Life technologies; Grand Island, NY). Following cDNA preparation with SuperScript IV Vilo (Thermofisher Scientific, Rockford, IL). Individual RT-PCR reactions were completed using a Bio-Rad CFX384 Touch Real-Time PCR thermal cycler, and TaqMan assays format. *TaqMan primer sets* were all from Thermofisher Scientific, Waltham MA: Lama1 (Mm01226102_m1), Lama2 (Mm00550083_m1), Lama3 (Mm01254735_m1), Lama4 (Mm01193660_m1), Lama5 (Mm01222029_m1), Lamb1 (Mm00801853_m1), Lamb2 (Mm00493080_m1), Lamb3 (Mm00493108_m1), Lamc1 (Mm00711820_m1), Lamc2 (Mm00500494_m1), Lamc3 (Mm01324510_m1), Lactase (Mm01276696_m1), Muc2 (Mm01276696_,1), Lysozyme (Mm00657323_m1), ChgA (Mm00514341_m1), Lgr5 (Mm00438890_m1), Olfm4 (Mm01320260_m1), mTert (Mm01352136_s1), HopX (Mm00558630_m1), NGN3 (Mm00437606_s1), Gata4 (Mm00437606_s1), Gata6 (Mm00802632_m1), Ihh (Mm00439613_m1), Ptchd1 (Mm00436026_m1), Abcg5 (Mm00433937_m1), Abcg8 (Mm00433937_m1), Ada (Mm00545720_m1), Akr1b8 (Mm00484314_m1), Apoa4 (Mm00431814_m1), ApoB (Mm00431814_m1), ApoC2 (Mm00433937_m1), ApoC3 (Mm00433937_m1), Asah2 (Mm00479659_m1), Cck (Mm00446170_m1), CD36 (Mm01135198_m1), Cftr (Mm00445197_m1), Dnase1 (Mm00445197_m1), Enpp7 (Mm01252812_m1), Fabp2 (Mm00433188_m1), Hsd17b6 (Mm00457343_m1), Pdx1 (Mm00435565_m1), Slc2a1 (Mm00441473_m1), Slc2a2 (Mm00441473_m1), Slc2a5 (Mm00433937_m1), Slc2a8 (Mm00433937_m1), Slc5a1 (Mm00433937_m1), Slc5a11 (Mm00433937_m1), Sst (Mm01436671_m1), and Ugt2a3 (Mm01436671_m1). Ct values were normalized against GAPDH (cat #4351309), also from Thermofisher Scientific, Walthan, MA. All samples were run as technical duplicates, with the number of biological replicates (n) indicated within the table.

### Statistical analysis

Data were analyzed using GraphPad Prism 7.0 software (La Jolla, CA) using the two-tailed students t-test. All data is represented as the mean ± SEM. Statistical significance was defined as a p-value of less than 0.05 in a cohort with no less than three biological replicates.

## Supplementary information

Supplementary Video 1

Supplementary Video 2

Supplementary Figures

## Data Availability

The datasets generated during and/or analyzed during the current study are available from the corresponding author on reasonable request.
