# Supplementary material for: The Adult Murine Intestine is Dependent on Constitutive Laminin-γ1 Synthesis
Source: Sci Rep. 2019 Dec 17;9:19303. doi: 10.1038/s41598-019-55844-x (PMC6917708; doi:10.1038/s41598-019-55844-x)

**Supplementary Data:**

**The Adult Murine Intestine is Dependent on Constitutive Laminin-γ1 Synthesis**

AUTHORS: British Fields^1*^, Ann DeLaForest^2*^, Mark Zogg^1^, Jennifer May^1^, Catherine Hagen^3^, Kristin Komnick^1^, Jon Wieser^1^, Alexander Lundberg^1^, Hartmut Weiler^1^, Michele A. Battle^2**^, Karen-Sue Carlson^1,4**^

INSTITUTIONAL AFFILIATION:

^1^The Blood Research Institute of Wisconsin, part of Versiti; Milwaukee, Wisconsin. ^2^The Medical College of Wisconsin, Department of Cell Biology, Neurobiology, and Anatomy; ^3^The Medical College of Wisconsin, Department of Pathology; ^4^The Medical College of Wisconsin, Department of Internal Medicine and Division of Hematology and Oncology; Milwaukee, Wisconsin.

*Co-first authorship

**Co-senior authorship

**Supplemental Figure 1**: Immunofluorescent analysis of laminin-α2 and laminin-α4 chains in control and laminin-γ1 depleted duodenum. The presented images are representative of three control and four laminin-γ1 depleted mice. In each instance, the magnification bar indicates 100microns.


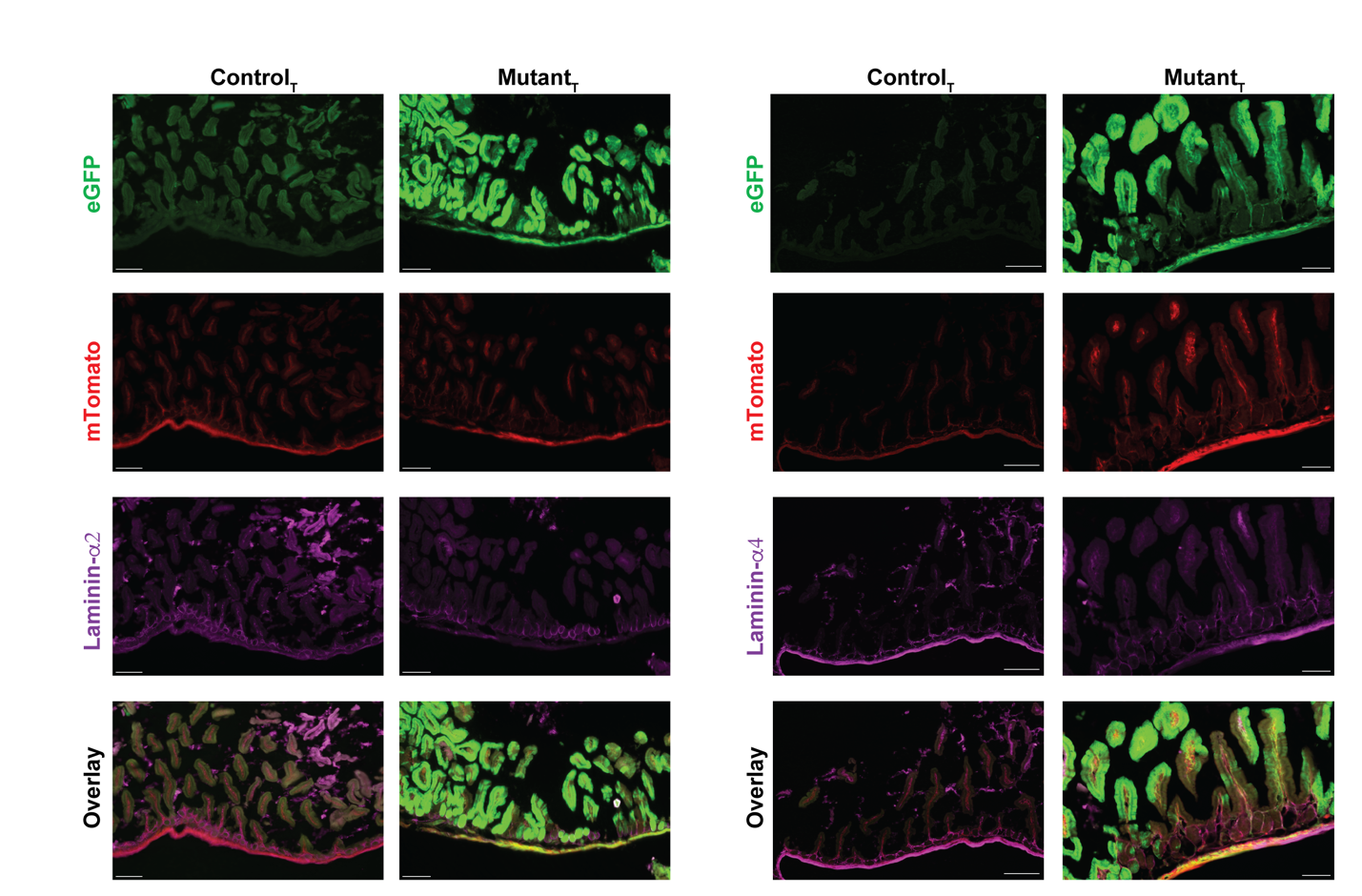


**Supplemental Figure 2**: Food and water intake following tamoxifen-induction. Food and water consumption were measured prior to and after tamoxifen administration to control and mutant mice. Each cohort began with 5 animals. On day 13, two mutant mice were euthanized based on morbidity criteria as detailed in our IACUC approved AUA. Two control animals were euthanized at that same time to facilitate paired measurements of gene recombination. On day 18, this study was concluded with two additional mutant mice meeting morbidity criteria for euthanasia. All animals were therefore euthanized at this timepoint. Each data point indicates results for an individual mouse.


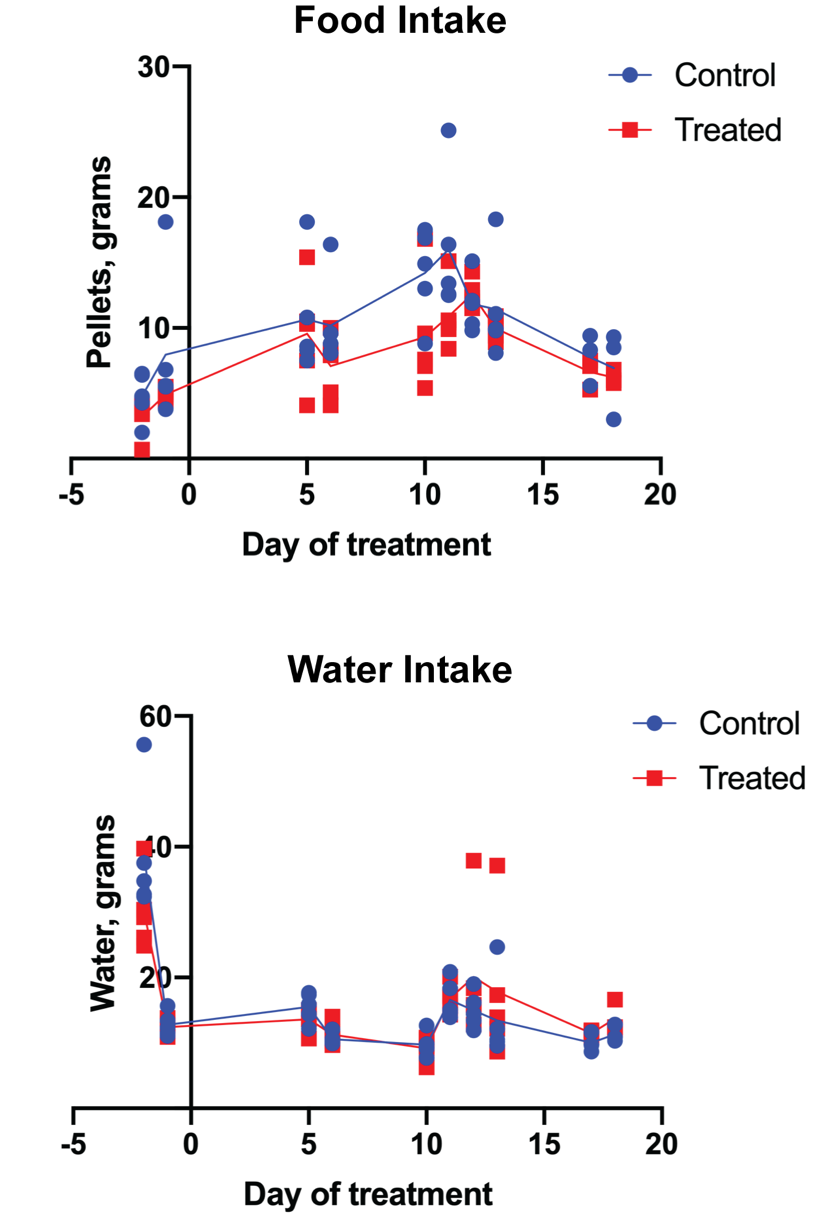


**Supplemental Figure 3**: Full western blot images for caspase 3 and B-tubulin.


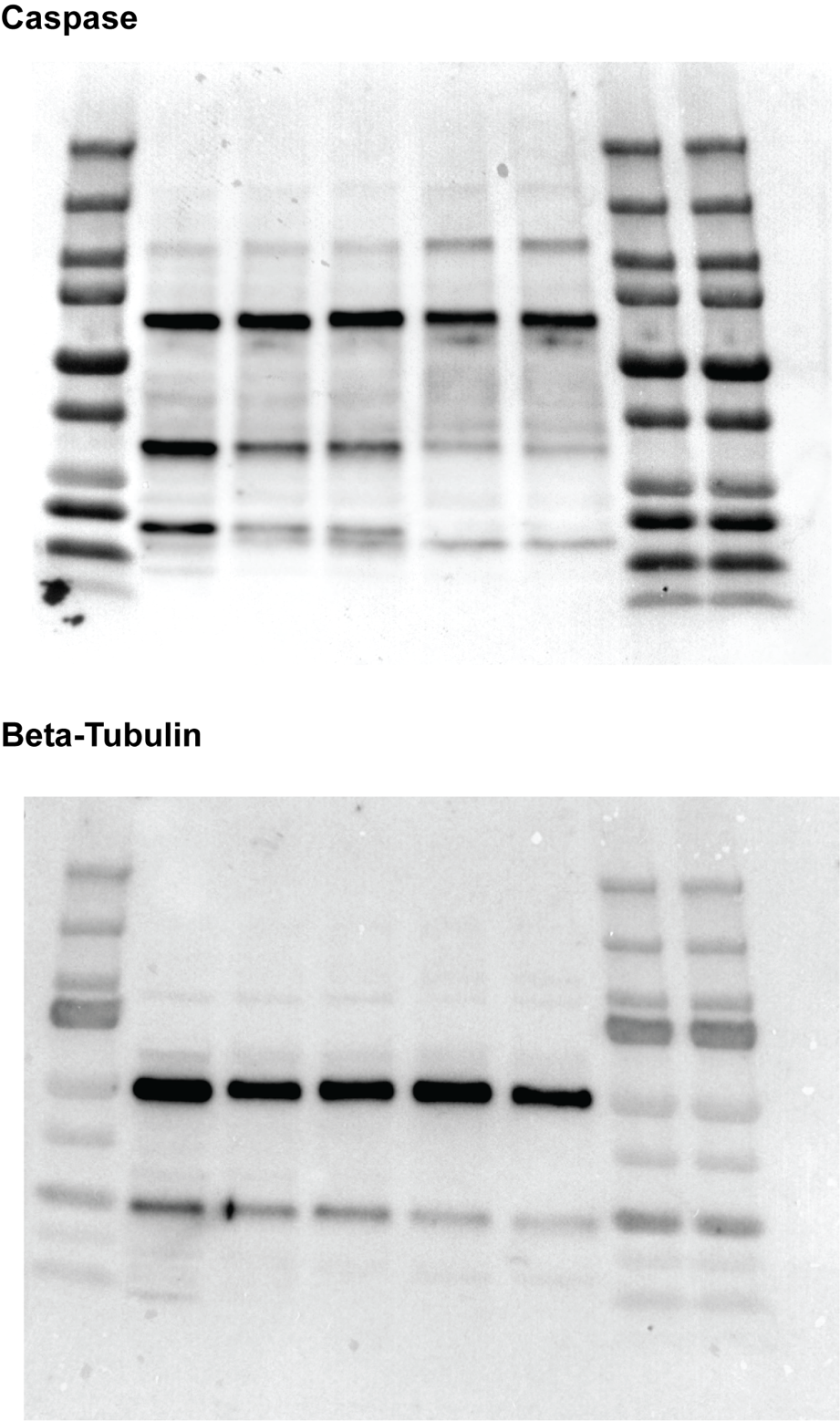

Supplement: Supplementary file 3 — Supplementary Figures [file 41598_2019_55844_MOESM3_ESM.docx]
